# Intravascular imaging-guided versus angiography-guided percutaneous coronary intervention: a reconstructed individual participant data meta-analysis of randomized controlled trials of intravascular ultrasound- and optical coherence tomography-guided strategies

**DOI:** 10.1093/ehjopen/oeag136

**Published:** 2026-09-10

**Authors:** Aref A Bin Abdulhak, Mamas A Mamas, Ziad A Ali, Roxana Mehran, Wayne B Batchelor, Razan Bahurmuz, Ghulam Mujtaba Ghumman, Ala Mohsen, Khawlah A Hadi, Anas Noman, Marwan Saad, Zaher Fanari, Hazim Rahbi, Musa Sharkawi, Poghni Peri-Okonny, Raveen Chawla, Mirvat Alasnag

**Affiliations:** Winchester Cardiology and Vascular Medicine, Winchester Medical Center, 1880 Amherst Street, Winchester, VA 22601, USA; Keele Cardiovascular Research Group, Centre for Prognosis Research, School of Medicine, David Weatherall Building, Keele University, Staffordshire ST5 5BG, UK; DeMatteis Cardiovascular Institute, St. Francis Hospital & Heart Center, 100 Port Washington Boulevard, Roslyn, NY 11576, USA; Zena and Michael A. Wiener Cardiovascular Institute, Icahn School of Medicine at Mount Sinai, One Gustave L. Levy Place, New York, NY 10029, USA; Inova Schar Heart and Vascular, Inova Health System, 3300 Gallows Road, Falls Church, VA 22042, USA; King Salman Heart Center, King Fahad Medical City, P.O. Box 13534, Riyadh 11525, Saudi Arabia; Department of Internal Medicine, Mercy St. Vincent Medical Center, 2213 Cherry Street, Toledo, OH 43608, USA; Department of Cardiology, Tulane University Hospital, 1430 Tulane Avenue, New Orleans, LA 70112, USA; East Jefferson Hospital, 4200 Houma Boulevard, Metairie, LA 70006, USA; Padra Clinic, 12 Blvrd, Anas Ibn Malik Street, Al Malqa District, Riyadh, Saudi Arabia; Department of Internal Medicine, Mercy St. Vincent Medical Center, 2213 Cherry Street, Toledo, OH 43608, USA; Brown University Health Cardiovascular Institute, 2 Dudley Street, Suite 360, Providence, RI 02905, USA; Division of Cardiology, UCSF Fresno, University of California San Francisco, 155 North Fresno Street, Fresno, CA 93701, USA; Heart Health Center, King Saud Medical City, Riyadh First Health Cluster, Sheikh Abdulaziz Bin Abdulrahman Al Saud Street, Al Shumaisi District, P.O. Box 780, Riyadh 12746, Saudi Arabia; Heart, Vascular, and Thoracic Institute, Cleveland Clinic Abu Dhabi, 59 Hamouda Bin Ali Al Dhaheri Street, Al Maryah Island, Abu Dhabi, United Arab Emirates; Winchester Cardiology and Vascular Medicine, Winchester Medical Center, 1880 Amherst Street, Winchester, VA 22601, USA; Winchester Cardiology and Vascular Medicine, Winchester Medical Center, 1880 Amherst Street, Winchester, VA 22601, USA; Cardiac Center, King Fahd Armed Forces Hospital, Al Kurnaysh Road, Jeddah 23311, Saudi Arabia

**Keywords:** IVUS, Angiography, OCT, meta-analysis

## Abstract

**Aims:**

Uncertainty persists regarding the comparative efficacy of intravascular ultrasound (IVUS) and optical coherence tomography (OCT) vs. angiography-guided percutaneous coronary intervention (PCI). We synthesized randomized evidence using reconstructed individual participant data (IPD) and compared the two modalities indirectly.

**Methods and results:**

MEDLINE and CENTRAL were searched up to 31 March 2026. IPD were reconstructed from published Kaplan–Meier curves. Primary analyses used one-stage stratified Cox models; secondary analyses included random-effects and Bayesian hierarchical models. The primary outcome was major adverse cardiovascular events (MACE). Twenty-three randomized controlled trials enrolling 21 176 patients were included (16 IVUS, *N* = 15 209; 7 OCT, *N* = 5967). IVUS-guided PCI reduced MACE [hazard ratio (HR) 0.74; 95% confidence interval (CI) 0.67–0.83; *P* < 0.001], target-vessel revascularization (TVR; HR 0.70; 0.60–0.82), and stent thrombosis (ST; HR 0.54; 0.37–0.78). OCT-guided PCI reduced MACE (HR 0.75; 0.63–0.91; *P* = 0.003), cardiac death (HR 0.48; 0.27–0.83), myocardial infarction (HR 0.79; 0.63–0.99), ST (HR 0.40; 0.22–0.74), and TVR (HR 0.74; 0.57–0.97). Bayesian posterior probabilities of benefit exceeded 82% for all IVUS and 94% for all OCT endpoints. Indirect comparison detected no significant between-modality difference. Meta-regression of IVUS trials revealed heterogeneity by geographic region (*P* < 0.001) and attenuation of benefit over time (β = +0.034/year; *P* = 0.004); OCT analyses were non-significant.

**Conclusion:**

Both IVUS and OCT reduced MACE, revascularization, and ST compared with angiography-guided PCI. These findings support intravascular imaging as a routine adjunct to PCI in the populations studied.

## Introduction

Angiography alone provides a two-dimensional silhouette of the coronary lumen and may underestimate plaque burden, stent underexpansion, and edge dissection.^1,2^ Intravascular imaging with intravascular ultrasound (IVUS) and optical coherence tomography (OCT) overcomes these limitations by providing high-resolution cross-sectional assessment of vessel anatomy, lesion morphology, and stent deployment and has been associated with improved clinical outcomes in multiple randomized trials.^3–8^ Nevertheless, individual trials are not powered for hard endpoints such as cardiac death and stent thrombosis (ST); the two most recent European randomized trials (IVUS-CHIP^9^ and OPTIMAL^10^) reported neutral point estimates, highlighting that the magnitude and consistency of benefit may vary by era, region, and the extent to which imaging findings are translated into procedural action. Direct randomized head-to-head comparisons of the two imaging modalities remain limited—most notably OCTIVUS,^11^ which demonstrated non-inferiority of OCT vs. IVUS for target-vessel failure at 1 year, leaving clinical decision-making largely reliant on indirect evidence.

The most comprehensive synthesis to date is the network meta-analysis by Stone *et al.*^12^ in Lancet, which pooled study-level aggregate data and reported significant reductions in target lesion failure, cardiac death, and ST with intravascular imaging guidance. Giacoppo *et al.*^13^ performed a frequentist and Bayesian pairwise meta-analysis of 24 randomized controlled trials (RCTs) with broadly concordant findings. While landmark, these analyses relied on published summary statistics rather than individual patient-level time-to-event data, and their searches closed before several recent trials that have since reshaped the evidence landscape.

We therefore performed the first reconstructed individual participant data (IPD) meta-analysis of all eligible RCTs comparing IVUS- or OCT-guided percutaneous coronary intervention (PCI) with angiography-guided PCI, following Preferred Reporting Items for Systematic Reviews and Meta-Analyses (PRISMA)-IPD reporting guidelines and using established IPD-reconstruction methodology.^14–16^ Our primary objective was to derive patient-level time-to-event estimates for major clinical outcomes using one-stage stratified Cox models; secondary objectives included Bayesian quantification of the probability of benefit, a pre-specified indirect comparison of IVUS vs. OCT through subgroup meta-regression using angiography as the common comparator, and meta-regressions on geographic region and year of publication to interrogate the sources of between-trial heterogeneity.

## Methods

### Literature search and study selection

This systematic review and reconstructed IPD meta-analysis was conducted according to the PRISMA 2020^15^ and PRISMA-IPD^16^ reporting guidelines. The protocol was registered on PROSPERO (CRD420261373850). MEDLINE and CENTRAL were searched from inception through 31 March 2026, without language restriction. Reference lists of included trials and recent systematic reviews were hand-searched; forward citation searching was also performed to identify additional eligible trials not captured by the primary database search. Eligible studies were RCTs comparing IVUS- or OCT-guided PCI with angiography-guided PCI in patients receiving at least one drug-eluting stent (DES), with at least one pre-specified clinical outcome. Trials comparing IVUS directly with OCT without an angiography-guided control arm were excluded. Two reviewers (A.A.B.A. and R.M.) independently extracted eligible studies from search results.

### Data extraction and risk of bias

Event counts per arm, hazard ratios (HRs) with 95% confidence intervals (CIs), and trial characteristics (population, DES type, follow-up, optimization criteria, and adjudication) were extracted and reviewed independently by three reviewers (R.B., A.M., and M.A.M.). Where HRs were not published, risk ratios (RR) were computed from raw event counts with Haldane–Anscombe correction^17^; where a *P*-value but no CI was reported, the CI was back-calculated. Risk of bias was assessed with the Cochrane Risk-of-Bias tool, version 2 (RoB 2.0).^18^ Two reviewers independently (A.A.B.A. and A.M.) assessed the quality of included studies.

### Outcomes

The primary outcome was each trial’s pre-specified primary ischaemic composite at the longest uniformly reported follow-up. Because individual trials used heterogeneous composite definitions (variously termed major adverse cardiovascular events or target-vessel failure), this outcome is referred to as ‘MACE’ throughout; the pooled estimate incorporates each trial’s own primary composite. Secondary outcomes were cardiac mortality, clinically driven target-lesion revascularization (TLR)/target-vessel revascularization (TVR), Academic Research Consortium-defined definite or probable ST, all-cause mortality, and—for the OCT cohort—myocardial infarction (MI) (captured within the primary composite in most IVUS trials).

### Statistical analysis

All statistical analyses were conducted in Python 3.12 (NumPy, SciPy, pandas, and matplotlib) using custom scripts executed under investigator supervision. Computational assistance from Claude (Anthropic, San Francisco, CA, USA) was used to support code generation and implementation; however, all analytic steps, model specifications, data inputs, and outputs were defined, reviewed, and verified by the investigators.

To ensure data integrity, all extracted data and reconstructed datasets were independently verified by three reviewers against the original trial reports (R.B., A.M., and M.A.M.).

To confirm validity and reproducibility, key analyses were independently replicated in a separate statistical environment (R, version 4.5.3; survival, meta, and metafor packages), reproducing similar HRs and CIs for the primary and key secondary endpoints, including both aggregate-data and reconstructed IPD analyses. This cross-platform validation ensured that results were not dependent on a specific software implementation.

Published Kaplan–Meier curves were digitized and individual time-to-event data reconstructed with the Guyot algorithm,^14^ anchored by reported numbers-at-risk and total events; reconstructed counts were proportionally rescaled as needed to match published totals while preserving curve shape. For endpoints where published Kaplan–Meier curves were not available or not reported, a parametric exponential approach was used: for each trial arm, survival times were generated using hazard rates estimated from published event counts and sample sizes, with follow-up durations matching reported trial protocols and event times calibrated to reproduce the exact number of events per arm. This constant-hazard parametric method, informed by the Guyot framework, preserves between-arm treatment effects while enabling Cox regression when only aggregate event counts are available. A one-stage Cox proportional hazards model stratified by trial was fit to each modality cohort (16 IVUS trials, *N* = 15 209; 7 OCT trials, *N* = 5967), with treatment as the sole covariate and Breslow tie-handling. Proportional hazards were assessed graphically and with the Grambsch–Therneau global score test.^19^

Robustness of findings was further assessed through multiple complementary analytical approaches, including one-stage stratified Cox proportional hazards models, two-stage random-effects meta-analysis using the DerSimonian–Laird (DL) estimator^20^ with Hartung–Knapp–Sidik–Jonkman CIs,^21^ and Bayesian hierarchical modelling. Heterogeneity was reported as I^22^ Cochran’s Q, and τ^2^.

To compare IVUS and OCT against the common angiography comparator, a subgroup meta-regression was fitted with modality as a binary moderator, following the Bucher framework adapted to the reconstructed IPD meta-analytic setting.

Pre-specified sensitivity analyses included leave-one-out analyses, restriction to trials at low risk of bias, exclusion of influential or large trials, and alternative model specifications. Additional sensitivity analyses evaluated the impact of trial era and geographic region, as well as inclusion of borderline or subgroup-derived datasets. Proportional hazards assumptions were assessed using Schoenfeld residuals, with time-restricted analyses performed where appropriate. Between-trial heterogeneity was reported as I^2^, Cochran’s Q, and τ^2^; small-study effects were examined with funnel plots and Egger’s test as exploratory analyses. All *P*-values are two-sided; *P* < 0.05 was considered statistically significant for the primary outcome, with no adjustment for multiplicity across the five secondary outcomes.

## Results

### Study characteristics

The systematic database search, supplemented by citation searching and reference list review (*Figure 1*), identified 23 RCTs enrolling 21 176 unique patients that met eligibility criteria: 16 IVUS trials [AVIO, HOME DES IVUS, Kim JS 2013, IVUS-XPL, CTO-IVUS, AIR-CTO, ULTIMATE, RENOVATE-COMPLEX-PCI (IVUS-predominant), IVUS-ACS, DKCRUSH-VIII, IVUS-CHIP, OPTIMAL, GUIDE-DES, Liu 2019, Tan 2015, and Bendary 2024; *N* = 15 209]^3–6,9,10,23–32^ and 7 OCT trials (DOCTORS, ILUMIEN IV, OCTOBER, OCCUPI, CALIPSO, OCTACS, and Kala 2018; *N* = 5967).^7,8,33–37^ The RENOVATE-COMPLEX-PCI OCT subgroup (278 patients) was excluded from the primary OCT analysis as a *post hoc* subgroup analysis but was included in a pre-specified sensitivity analysis. Trials were conducted between 2009 and 2026; median follow-up was 24 months (range 1–60). Complex lesion subsets [bifurcation, chronic total occlusion (CTO), left main, and long lesions] comprised the majority of enrolled patients. Per-trial pre-specified ischaemic composite endpoints and individual component definitions (MACE, cardiac death, MI, TVR/TLR, and ST) are detailed in *Table 1*. All trials were open-label due to the unblindable nature of intravascular imaging, although independent blinded outcome adjudication was present in 15 of 23 trials. Risk of bias was low in 14 trials and raised some concerns in 9; baseline trial characteristics are summarized in *Table 1*.

**Figure 1:**
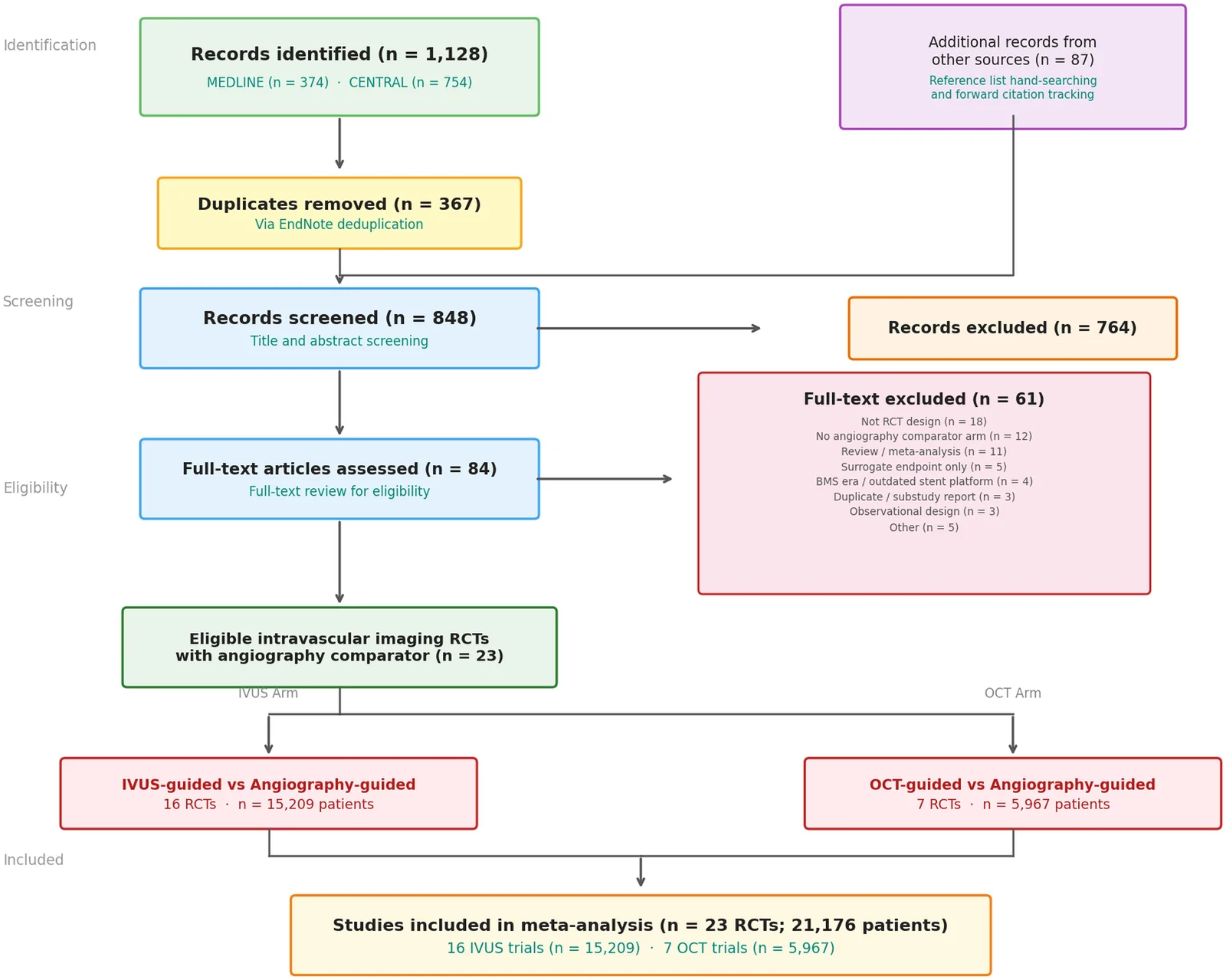
Flow diagram of included studies.

**Table 1:** Characteristics of included randomized trials

Modality	Trial	Year	*N*	Population	Follow-up, months
**IVUS**	AVIO^23^	2013	284	Complex	24
**IVUS**	IVUS-XPL^3^	2015	1400	Long lesions	60
**IVUS**	CTO-IVUS^24^	2015	402	CTO	12
**IVUS**	ULTIMATE^4^	2018	1448	All-comers	36
**IVUS**	RENOVATE-COMPLEX-PCI (IVUS-predominant)^5^	2023	1639	Complex	24
**IVUS**	IVUS-ACS^6^	2024	3505	ACS	12
**IVUS**	DKCRUSH-VIII^25^	2026	555	Bifurcation	12
**IVUS**	IVUS-CHIP^9^	2026	2019	Complex/high-risk	12
**IVUS**	OPTIMAL^10^	2026	806	Left main/complex	12
**IVUS**	GUIDE-DES^26^	2024	1528	All-comers (QCA vs. IVUS)	12
**IVUS**	Liu 2019^27^	2019	336	Unprotected LMCA	12
**IVUS**	Tan 2015^28^	2015	123	Elderly LMCA	24
**IVUS**	Kim JS 2013^29^	2013	543	Long lesions	12
**IVUS**	HOME DES IVUS^30^	2010	210	Complex lesions (B2/C)	18
**IVUS**	AIR-CTO^31^	2015	230	CTO lesions	24
**IVUS**	Bendary 2024^32^	2024	181	Left main	12
**OCT**	DOCTORS^33^	2016	240	NSTEMI	6
**OCT**	ILUMIEN IV^7^	2023	2487	Complex/high-risk	24
**OCT**	OCTOBER^8^	2023	1201	Bifurcation	24
**OCT**	OCCUPI^34^	2024	1604	Complex	12
**OCT**	CALIPSO^35^	2025	134	Calcified	1
**OCT**	OCTACS^36^	2015	100	NSTE-ACS	6
**OCT**	Kala 2018^37^	2018	201	STEMI	9

ACS, acute coronary syndrome; CTO, chronic total occlusion; IVUS, intravascular ultrasound; LMCA, left main coronary artery; *N*, number of patients; NSTE-ACS, non-ST-elevation acute coronary syndrome; NSTEMI, non-ST-elevation myocardial infarction; OCT, optical coherence tomography; QCA, quantitative coronary angiography; STEMI, ST-elevation myocardial infarction.

### Intravascular ultrasound-guided percutaneous coronary intervention versus angiography

In the one-stage stratified Cox reconstructed IPD analysis of 16 IVUS trials (*N* = 15 209), IVUS-guided PCI significantly reduced MACE (HR 0.74; 95% CI 0.67–0.83; *P* < 0.001), TVR/TLR (HR 0.70; 95% CI 0.60–0.82; *P* < 0.001), and definite/probable ST (HR 0.54; 95% CI 0.37–0.78; *P* = 0.001). Cardiac death reduction did not reach statistical significance (HR 0.79; 95% CI 0.62–1.01; *P* = 0.06), nor did all-cause mortality (HR 0.85; 95% CI 0.70–1.03; *P* = 0.09). Two-stage DL random-effects estimates were directionally concordant but larger in magnitude with higher between-trial heterogeneity (MACE HR 0.67; 95% CI 0.54–0.83; I^2^ = 72%; TVR/TLR HR 0.65; 95% CI 0.51–0.82; I^2^ = 44%). Bayesian hierarchical estimates yielded posterior probabilities of benefit of 99.3% for MACE, 98.4% for TVR/TLR, 89.2% for ST, 86.3% for cardiac death, and 82.5% for all-cause mortality (*Figures 2* and *3*, and Supplementary material online, *Table S1*).

**Figure 2:**
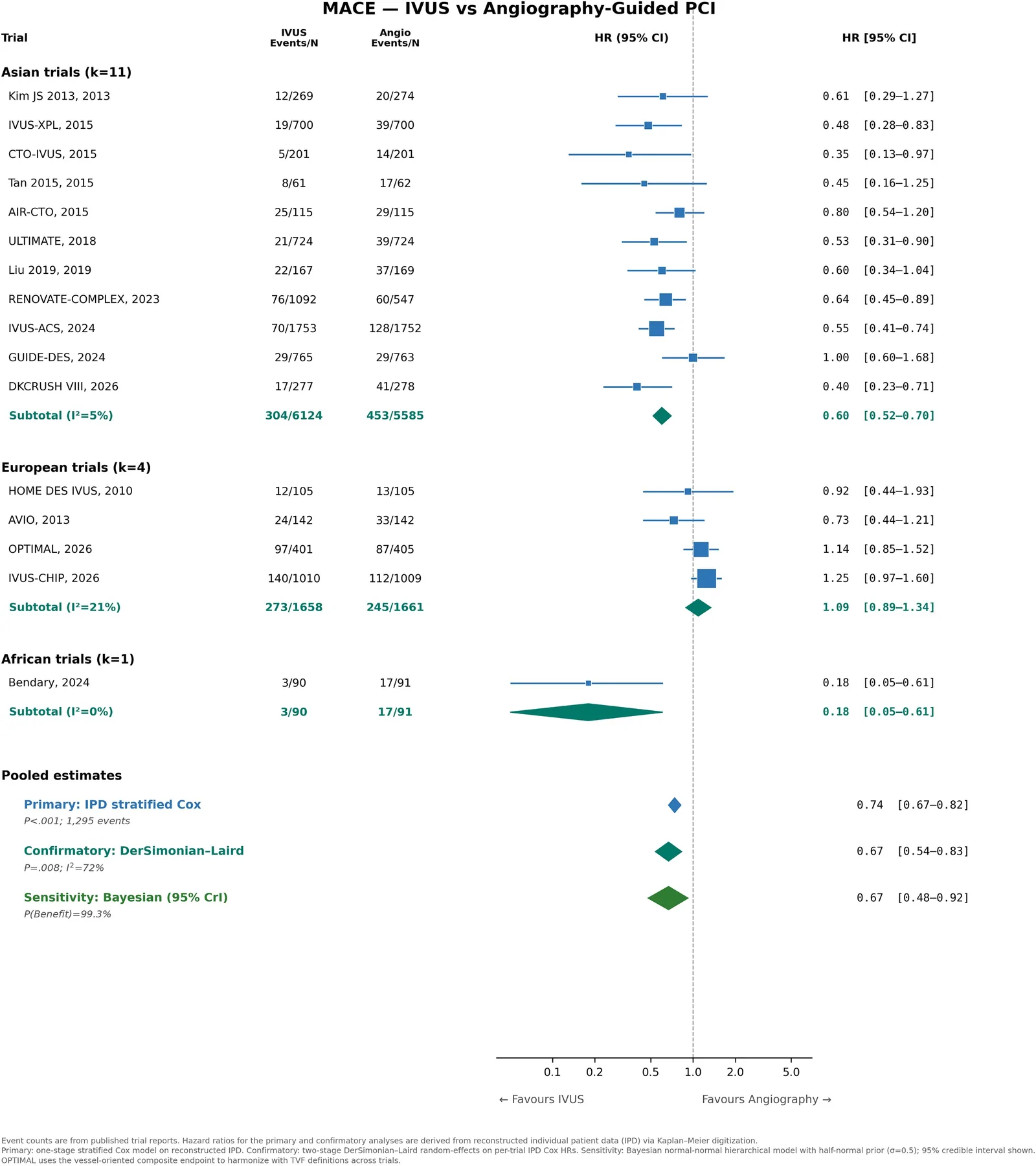
Forest plot of intravascular ultrasound-guided vs. angiography-guided percutaneous coronary intervention for the primary composite endpoint (major adverse cardiovascular events) across 16 randomized trials (*N* = 15 209). Per-trial hazard ratios were derived from Guyot-reconstructed individual participant data via Kaplan–Meier digitization; event counts are from published trial reports. Trials are stratified by geographic region (Asian, *K* = 11; European, *K* = 4; African, *K* = 1). Pooled estimates are shown for the primary analysis (one-stage stratified Cox model on reconstructed individual participant data), the confirmatory analysis (two-stage DerSimonian–Laird random-effects on per-trial individual participant data Cox hazard ratios), and a sensitivity analysis [Bayesian normal–normal hierarchical model with half-normal prior (σ = 0.5); 95% credible interval shown]. Square size is proportional to inverse-variance weight; diamonds represent subgroup and pooled estimates with 95% confidence intervals.

**Figure 3:**
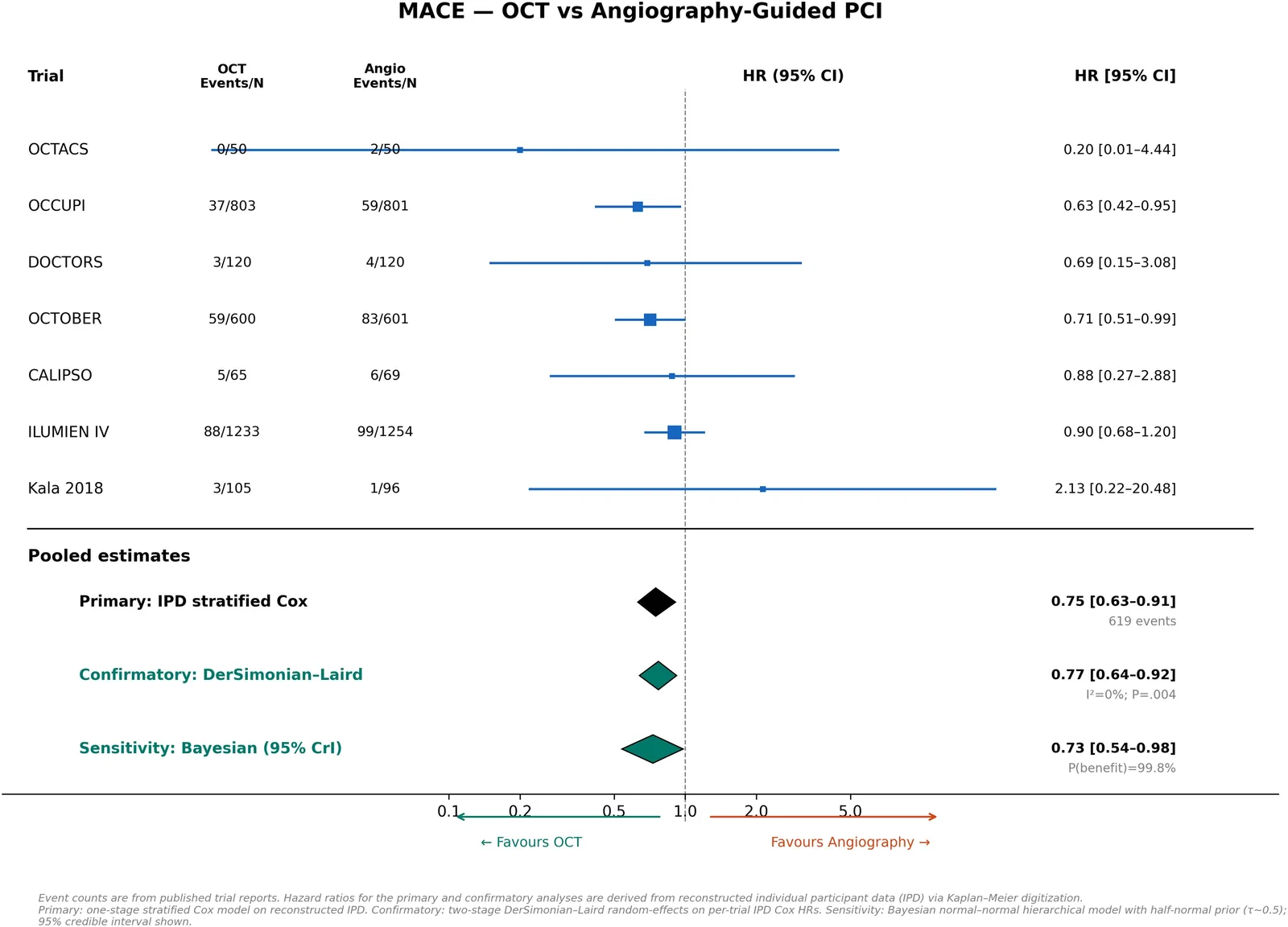
Bayesian posterior density plots comparing imaging-guided (IVUS, solid lines; OCT, dashed lines) versus angiography-guided PCI across all five prespecified outcomes. For each endpoint the shaded area to the left of relative risk (RR)= 1.0 represents the posterior probability of benefit; annotated percentages quantify this probability for each modality. Square size is proportional to inverse-variance weight; diamonds represent pooled estimates with 95% confidence or credible intervals shown

### Optical coherence tomography-guided percutaneous coronary intervention versus angiography

In the one-stage stratified Cox reconstructed IPD analysis of seven OCT trials (5967 patients), OCT-guided PCI significantly reduced MACE (HR 0.75; 95% CI 0.63–0.91; *P* = 0.003), cardiac death (HR 0.48; 95% CI 0.27–0.83; *P* = 0.009), MI (HR 0.79; 95% CI 0.63–0.99; *P* = 0.038), and ST (HR 0.40; 95% CI 0.22–0.74; *P* = 0.004), with significant reduction in TVR (HR 0.74; 95% CI 0.57–0.97; *P* = 0.029). DL random-effects estimates were concordant for MACE (RR 0.77; *P* = 0.004), cardiac death (RR 0.56; *P* = 0.037), and ST (RR 0.49; *P* = 0.019); MI showed a borderline trend (RR 0.81; *P* = 0.059) and TVR showed moderate heterogeneity (I^2^ = 54%; *P* = 0.129). Bayesian posterior probability of benefit was 99.8% for MACE, 99.2% for cardiac death, 97.2% for MI, 99.6% for ST, and 94.4% for TVR (*Figures 3* and *4*). A sensitivity analysis including the RENOVATE-COMPLEX-PCI OCT subgroup (8 trials, 6271 patients) demonstrated significance for all five endpoints.

**Figure 4:**
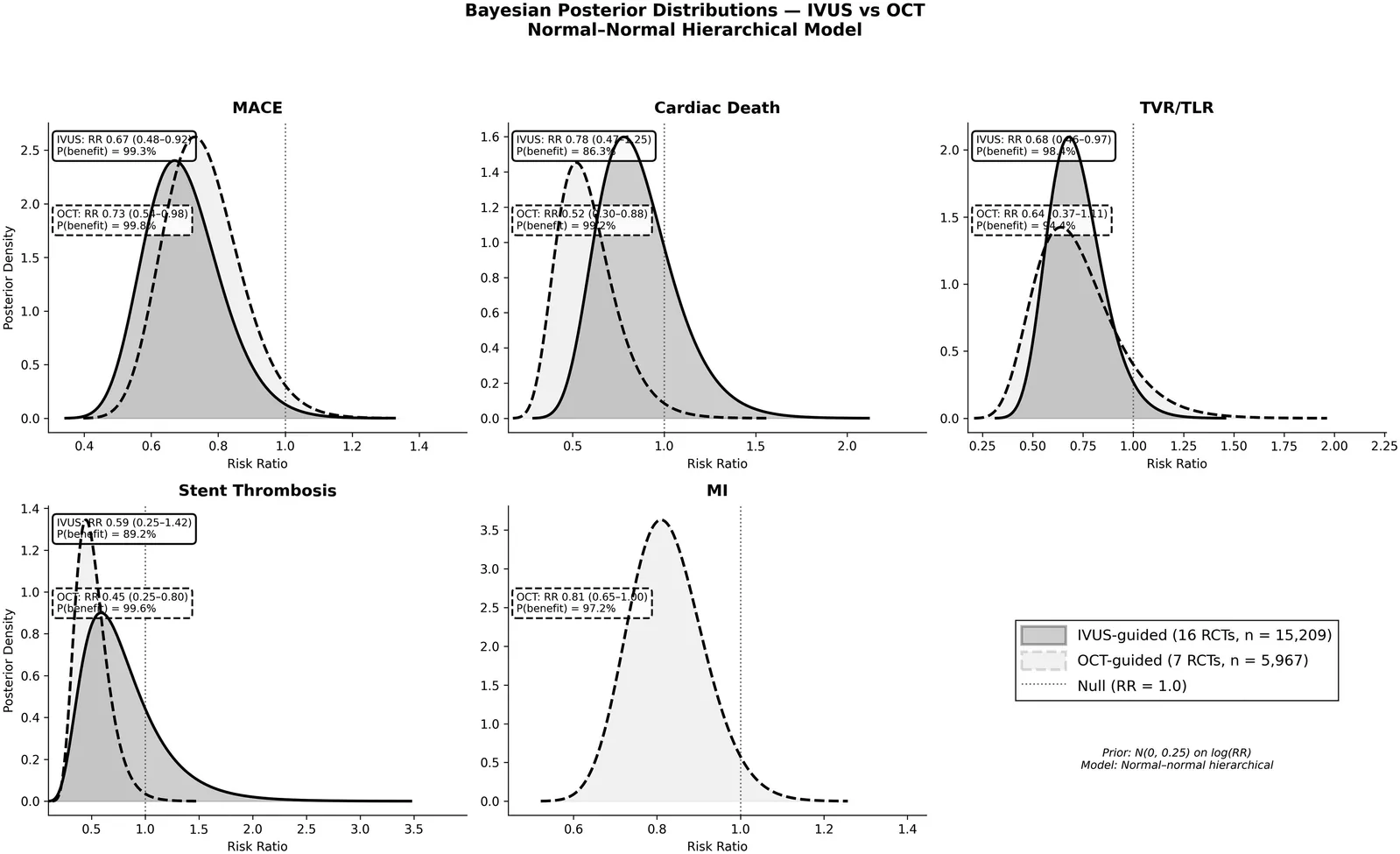
Forest plot of optical coherence tomography-guided vs. angiography-guided percutaneous coronary intervention for the primary composite endpoint (major adverse cardiovascular events) across seven randomized trials (*N* = 5967). Per-trial hazard ratios were derived from Guyot-reconstructed individual participant data via Kaplan–Meier digitization; event counts are from published trial reports. Pooled estimates are shown for the primary analysis (one-stage stratified Cox model on reconstructed individual participant data), the confirmatory analysis (two-stage DerSimonian–Laird random-effects on per-trial individual participant data Cox hazard ratios), and a sensitivity analysis [Bayesian normal–normal hierarchical model with half-normal prior (σ = 0.5); 95% credible interval shown]. Square size is proportional to inverse-variance weight; diamonds represent pooled estimates with 95% confidence or credible intervals.

### Indirect comparison of intravascular ultrasound and optical coherence tomography

Because very few RCTs have randomized patients directly between IVUS and OCT, we performed an indirect comparison via subgroup meta-regression using angiography-guided PCI as the common comparator, with imaging modality (IVUS vs. OCT) entered as a binary moderator on pooled log-HRs. This approach tests whether the effect of intravascular imaging vs. angiography differs between IVUS trials and OCT trials; it does not constitute a within-trial randomized comparison of the two modalities. No statistically significant between-modality difference was detected for any outcome with overlapping reconstructed IPD estimates, MACE (*P* for interaction=0.88), cardiac death (*P* = 0.06, with a larger point estimate of benefit for OCT), ST (*P* = 0.55), and TVR/TLR (*P* = 0.94). MI was a pre-specified reconstructed IPD outcome in the OCT dataset only; the IVUS trials did not uniformly report MI as a harmonized time-to-event endpoint in the reconstructed dataset and was therefore not included in the direct between-modality comparison. Pooled across modalities, intravascular imaging reduced MACE by approximately 25% (HR 0.75; 95% CI 0.68–0.82; *P* < 0.001) with minimal heterogeneity by modality. These findings are directionally consistent with the only adequately powered direct randomized head-to-head trial to date (OCTIVUS; *n*≈2000), which reported non-inferiority of OCT vs. IVUS for target-vessel failure at 1 year (see Supplementary material online, *Table S1*).

### Heterogeneity by region and year of publication

Because between-trial heterogeneity was more prominent for IVUS than for OCT, two pre-specified meta-regressions were performed on the IVUS dataset with geographic region and year of publication as moderators. In the 11 Asian IVUS trials (Kim JS 2013, AIR-CTO, CTO-IVUS, IVUS-XPL, Tan 2015, ULTIMATE, Liu 2019, RENOVATE-COMPLEX-PCI, GUIDE-DES, IVUS-ACS, DKCRUSH-VIII; *N* = 11 709), IVUS-guided PCI reduced MACE with a pooled HR of 0.60 (95% CI 0.51–0.70; I^2^ = 11%). In the four European trials (HOME DES IVUS, AVIO, IVUS-CHIP, and OPTIMAL; *N* = 3319), the pooled HR was 1.11 (95% CI 0.91–1.35; I^2^ = 15%), with IVUS-CHIP (HR 1.25) and OPTIMAL (HR 1.14) showing point estimates above unity while AVIO (HR 0.73) and HOME DES IVUS (HR 0.92) favoured IVUS. The single African trial (Bendary 2024; *N* = 181) strongly favoured IVUS (HR 0.18). The between-region difference was statistically significant (*P* for interaction<0.001). A univariate meta-regression on year of publication demonstrated attenuation of the IVUS effect over time (β = +0.034 per year on the log-HR scale; *P* = 0.004). A sensitivity analysis restricted to the thirteen trials published before 2026 (excluding DKCRUSH-VIII, IVUS-CHIP, and OPTIMAL) yielded a pooled HR of 0.63 (95% CI 0.55–0.73) with I^2^ = 0%. Corresponding analyses within the OCT dataset were non-significant (*P* for region interaction = 0.76; *P* for year=0.84).

### Sensitivity and bias assessment

The overall risk of bias, assessed with the Cochrane RoB 2.0 tool, was low in 14 of the 23 included trials (AVIO, IVUS-XPL, ULTIMATE, RENOVATE, GUIDE-DES, IVUS-ACS, IVUS-CHIP, OPTIMAL, DOCTORS, Kala 2018, ILUMIEN IV, OCTOBER, OCCUPI, and CALIPSO) and raised some concerns in 9. Among the 9 trials with some concerns, the most common sources were the deviations-from-intended-intervention and measurement-of-outcome domains (all trials were open-label due to the nature of the intervention, although independent blinded outcome adjudication was present in 15 of 23 trials), as shown in the traffic-light summary (*Figure 5*). Results were robust across sensitivity analyses: leave-one-out (see Supplementary material online, *Table S4*), restriction to low-risk-of-bias trials, exclusion of the largest trials, and two-stage reconstructed IPD with restricted maximum likelihood (REML) + Hartung–Knapp–Sidik–Jonkman (HKSJ) all produced concordant estimates (see Supplementary material online, *Table S4*). Funnel plots (see Supplementary material online, *Figure S1*) and Egger’s test showed no evidence of small-study effects in either the IVUS or OCT set. Bayesian posterior estimates were robust to alternative prior specifications (*Figure 3*).

**Figure 5:**
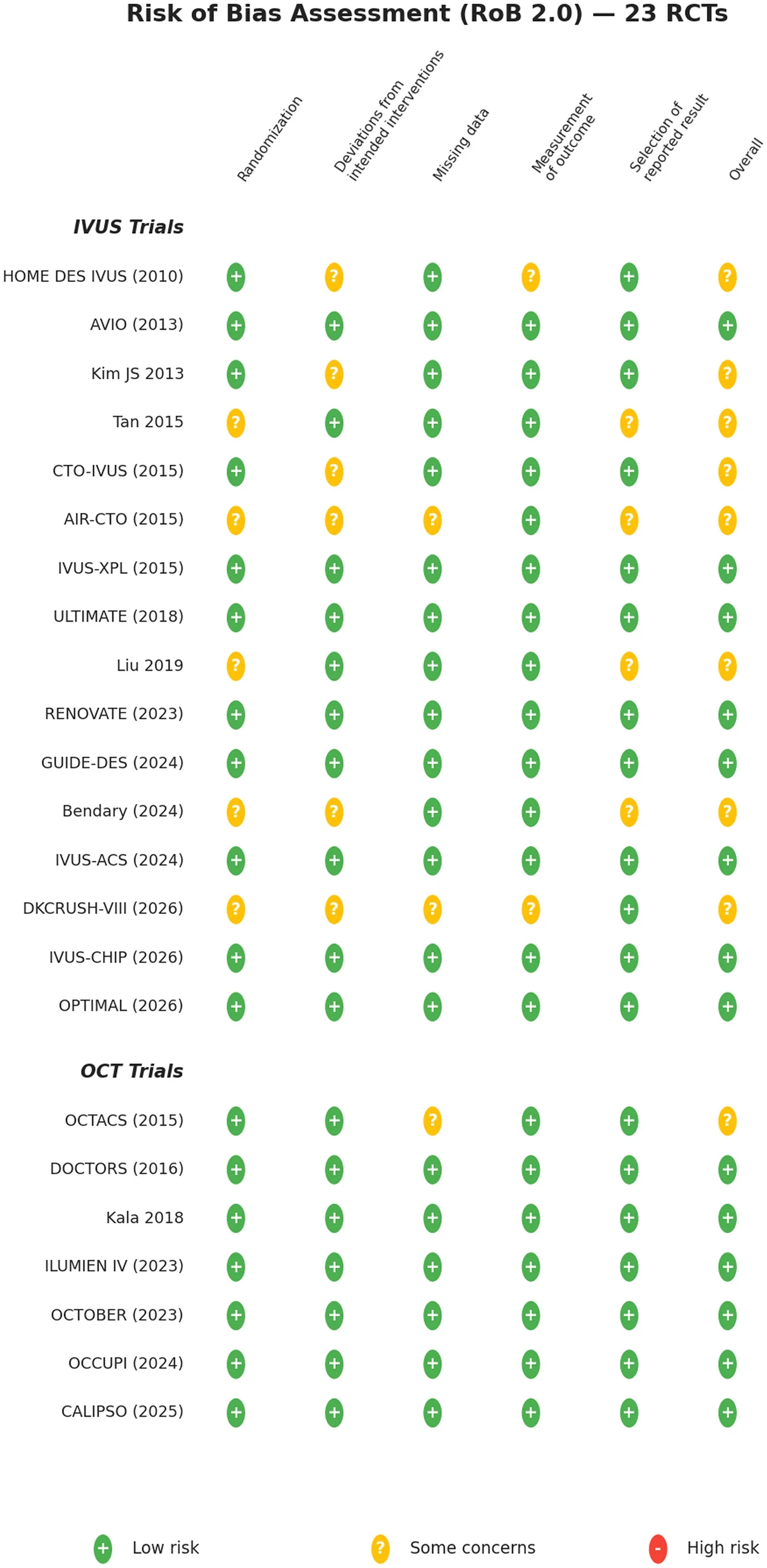
Cochrane Risk-of-Bias tool, version 2.0 traffic-light summary for all 23 included randomized trials across the five risk-of-bias domains.

## Discussion

This reconstructed IPD meta-analysis of 23 RCTs enrolling 21 176 patients demonstrates that both IVUS- and OCT-guided PCI significantly reduce MACE, repeat revascularization, and ST compared with angiography-guided PCI. OCT-guided PCI additionally reduced cardiac death (HR 0.48; *P* = 0.009), MI (HR 0.79; *P* = 0.038), and ST (HR 0.40; *P* = 0.004); cardiac death reduction did not reach statistical significance with IVUS (HR 0.79; 95% CI 0.62–1.01; *P* = 0.06), nor did all-cause mortality (HR 0.85; 95% CI 0.70–1.03; *P* = 0.09). The indirect comparison between modalities identified no statistically significant difference across any pre-specified outcome, consistent with findings from OCTIVUS, the largest direct randomized head-to-head comparison of the two modalities to date.

The pooled MACE reduction of approximately 25% with both IVUS and OCT, together with a roughly halved rate of ST across both modalities, is consistent with mechanisms of optimized stent sizing, expansion, and landing-zone selection. The reduction in cardiac death with OCT (HR 0.48; Bayesian posterior probability 99.2%) was statistically significant (*P* = 0.009), whereas cardiac death reduction did not reach significance with IVUS (HR 0.79; 95% CI 0.62–1.01; *P* = 0.06), a finding that may reflect differences in trial populations and follow-up duration and warrants investigation in future trials. One-stage reconstructed IPD and two-stage DL random-effects estimates were directionally concordant for all endpoints; the reconstructed IPD one-stage stratified Cox estimate, which preserves patient-level time-to-event information, is regarded as the primary inference.

The heterogeneity observed within the IVUS trial set is a central finding requiring careful interpretation. The 13 IVUS trials published before 2026 produced consistent benefit with a pooled HR of 0.63 (95% CI 0.55–0.73; I^2^ = 0%), whereas the 3 2026 trials (DKCRUSH-VIII, IVUS-CHIP, and OPTIMAL) showed mixed results: DKCRUSH-VIII (HR 0.40) strongly favoured IVUS, while IVUS-CHIP (HR 1.25) and OPTIMAL (HR 1.14) showed neutral point estimates. Several non-exclusive hypotheses may account for this discrepancy, none of which can be confirmed from the available data. First, it is possible that angiography-guided PCI technique has improved over time through greater use of appropriately sized stents, routine high-pressure post-dilation, and more systematic lesion preparation, narrowing the incremental benefit of imaging guidance. This hypothesis is supported by the significant year-of-publication meta-regression (β=+0.034 per year on the log-HR scale; *P* = 0.004). Second, the European trials enrolled particularly challenging substrates—severe calcification in IVUS-CHIP and left-main disease in OPTIMAL—where anatomic complexity may limit the degree to which imaging-derived information can be translated into procedural optimization. Third, differences in operator volume, catheterization-laboratory workflow, and geographic practice patterns may independently influence imaging utilization and its clinical impact.

A corollary is that the benefit of intravascular imaging appears to be concentrated in lesions where pre-specified criteria for optimal stent expansion, apposition, and edge coverage are actually achieved. Prior meta-analyses, including work by Elgendy *et al*.,^38^ have demonstrated that the clinical benefit of IVUS-guided PCI is driven primarily by reduced TLR when imaging findings are translated into procedural optimization. In IVUS-CHIP, optimization criteria were met in only 48% of analysable lesions. Importantly, the inability to achieve optimization thresholds may reflect lesion-related constraints rather than operator shortcomings where in heavily calcified or fibrotic lesions, plaque redistribution behind the stent has finite anatomic limits, and additional post-dilation beyond a certain point risks perforation or dissection without meaningful gain in stent area. A formal meta-regression by proportion of lesions achieving optimal imaging criteria was not performed in the present analysis because optimization was neither uniformly defined nor consistently reported across trials; future studies should address this gap. Nevertheless, this observation reframes the question for future trials where the intervention of interest is not imaging acquisition *per se*, but imaging-directed procedural action, and the achievability of optimization targets may itself vary by lesion complexity. Trial designs that mandate and verify achievement of pre-specified optimization thresholds—and that stratify outcomes by optimization achieved vs. not achieved are needed to disentangle these effects.

Taken together, these findings suggest that the incremental benefit of intravascular imaging over angiography is not fixed but varies by era, geography, and the baseline quality of the angiography-guided comparator. As contemporary angiographic technique improves, the measurable margin for imaging-guided gains narrows, a pattern reflected in the significant year-of-publication meta-regression and the geographic heterogeneity of the IVUS dataset. The absence of a significant difference between IVUS and OCT across all pre-specified outcomes is consistent with a class effect of imaging-guided PCI optimization rather than a modality-specific advantage, and implies that the choice between IVUS and OCT can be guided by clinical context including lesion anatomy, operator expertise, and catheterization-laboratory availability—rather than by expected differences in hard outcomes. However, this comparison is indirect and should be interpreted with caution, as differences in trial era (earlier IVUS trials vs. more recent OCT trials), DES technology, and patient populations (the IVUS evidence base includes more left main coronary artery (LMCA) and CTO cases, while OCT trials predominantly enrolled non-LMCA lesions) may confound the comparison. Perhaps most importantly, the frontier of this field is no longer whether to image but how to ensure that imaging-derived information is consistently translated into procedural optimization. Future trials should mandate achievement of pre-specified optimization targets and stratify outcomes by optimization achieved vs. not achieved, recognizing that the ceiling for optimization may itself vary by lesion complexity.

The neutral findings of the two most recent European IVUS trials deserve specific attention. IVUS-CHIP enrolled patients with severely calcified lesions and reported an HR of 1.25 for the primary composite, with optimization criteria met in only 48% of analysable lesions. OPTIMAL enrolled a broad spectrum of left-main disease and reported an HR of 1.14. Several factors may explain these results. First, the quality of angiography-guided PCI in contemporary European practice may be higher than in earlier East Asian trials, with more routine high-pressure post-dilation and systematic lesion preparation, leaving less room for incremental benefit from imaging. This is reflected in the lower absolute event rates in the angiography arms of these trials. Second, the degree to which IVUS findings led to changes in procedural strategy may have been suboptimal in both trials. Third, the specific lesion substrates enrolled—severe calcification in IVUS-CHIP, complex left-main disease in OPTIMAL—may impose anatomic ceilings on achievable optimization. Importantly, while neither trial reached statistical significance individually, their point estimates are not inconsistent with the pooled effect size observed in our meta-analysis, suggesting that these trials may have been individually underpowered for a composite endpoint with low event rates rather than genuinely contradictory.

The quality of intravascular imaging utilization itself warrants scrutiny as the quality and completeness of imaging-guided optimization also varied across trials. OCCUPI demonstrated the highest rates of optimization achievement using a comprehensive set of OCT-specific criteria and showed one of the largest magnitudes of benefit (HR 0.63 for MACE), raising the possibility that more rigorous optimization protocols yield greater clinical returns. Data from ILUMIEN IV, OCTOBER, and RENOVATE-COMPLEX on optimization achievement rates show variable criterion fulfilment across trials.

Geographic heterogeneity in the treatment effect of intravascular imaging is an important finding of this analysis. The IVUS benefit was substantially more pronounced in East Asian trials than in European trials (Asian subtotal HR 0.60 vs. European subtotal HR 1.09; *P* for interaction <0.001). South Korea in particular has contributed several of the most impactful studies (RENOVATE-COMPLEX and OCCUPI), and practice patterns unique to that region including high operator volume, established Intravascular imaging (IVI) workflows, and a healthcare system that facilitates imaging adoption, may contribute to the larger treatment effects observed. Our IPD approach adds time-to-event resolution to this geographic interaction, and the attenuation of benefit over time (meta-regression β = +0.034 per year; *P* = 0.004) suggests that secular improvements in angiography-guided technique may be narrowing the gap.

Two specific trials are worth further discussion. RENOVATE-COMPLEX trial randomized patients to an intravascular imaging-guided arm (where the modality was chosen at operator discretion with approximately 75% IVUS and 25% OCT) vs. angiography guidance. We classified this trial under IVUS in the primary analysis given the predominant modality used, while including the OCT subgroup (278 patients) in a pre-specified sensitivity analysis. The OCTOBER trial permitted IVUS use for left main lesions in the angiography-guided arm per protocol, and approximately half of LMCA patients in the control arm received IVUS guidance, representing an overall crossover rate of approximately 12% of control-arm patients. OCTOBER was analysed by intention-to-treat, consistent with its published primary analysis. This crossover would be expected to bias results towards the null, potentially underestimating the true benefit of intravascular imaging guidance. Despite this conservative bias, OCT-guided PCI demonstrated significant benefit in the pooled analysis.

Several limitations merit consideration, including reliance on reconstructed rather than true patient-level data where published Kaplan–Meier curves were available, digitized curves were reconstructed with the Guyot algorithm; where curves were unavailable, a parametric exponential model calibrated to published event counts was used, which does not capture the exact shape of each trial’s survival curve. Reconstructed IPD does not permit adjustment for patient-level covariates (e.g. diabetes, lesion complexity, calcification) beyond what is captured in the trial-level stratification; true patient-level subgroup analyses by lesion type (LMCA, calcification, and in-stent restenosis) would require a collaborative IPD meta-analysis using original trial datasets. The primary endpoint relied on trial-specific MACE definitions, which differed across studies (see Supplementary material online, *Table S4*); while our secondary analyses of individual hard endpoints are not subject to this concern, the heterogeneity of composite definitions is an inherent limitation of the primary analysis. Importantly, the comparison between IVUS and OCT in this analysis is indirect and derived from subgroup meta-regression across trials that used angiography as the common comparator rather than from within-trial randomization of patients to the two modalities. Such indirect comparisons are susceptible to between-trial differences in patient populations, lesion complexity, operator experience, and era, and yield wider uncertainty than within-trial randomized data. Additional limitations include variability in operator experience and imaging protocols and residual heterogeneity in complex lesion definitions.

## Conclusions

In this reconstructed IPD meta-analysis of 23 RCTs, both IVUS- and OCT-guided PCI significantly reduced MACE, ST, and repeat revascularization compared with angiography-guided PCI. OCT-guided PCI additionally reduced cardiac death, MI, and ST, while IVUS showed directionally consistent but non-significant mortality trends. These findings support intravascular imaging as a routine adjunct to contemporary PCI practice within the context of the population studied in the included trials, and underscore the need for investments in operator training, catheterization-laboratory workflow, and reimbursement infrastructure to ensure that imaging-derived information is consistently translated into procedural optimization.

## Supplementary Material

oeag136_Supplementary_Data

## Data Availability

Data generated for this study will be shared upon reasonable request to the corresponding author.
