# Supplementary material for: Intravascular imaging-guided versus angiography-guided percutaneous coronary intervention: a reconstructed individual participant data meta-analysis of randomized controlled trials of intravascular ultrasound- and optical coherence tomography-guided strategies
Source: Eur Heart J Open. 2026 Sep 10;6(5):oeag136. doi: 10.1093/ehjopen/oeag136 (PMC13717910; doi:10.1093/ehjopen/oeag136)
Supplement: Supplementary file 1 [file oeag136_supplementary_data.docx]

# Supplementary Material

Intravascular Ultrasound and Optical Coherence Tomography versus Angiography-Guided Percutaneous Coronary Intervention: A Reconstructed Individual Participant Data and Bayesian Network Meta-Analysis of Randomized Controlled Trials

## Supplementary Table 1. Outcomes by modality — reconstructed IPD (Cox) and DL random-effects results

| Modality | Outcome | Analysis | Trials | HR/RR (95% CI) | P value | I² |
| --- | --- | --- | --- | --- | --- | --- |
| IVUS | MACE | IPD (Cox) | 16 | 0.74 (0.67–0.83) | <.001 | — |
| IVUS | MACE | DL aggregate | 16 | 0.67 (0.54–0.83) | <.001 | 72% |
| IVUS | Cardiac Death | IPD (Cox) | 16 | 0.79 (0.62–1.01) | .07 | — |
| IVUS | Cardiac Death | DL aggregate | 16 | 0.73 (0.53–1.00) | .050 | 20% |
| IVUS | All-Cause Mortality | IPD (Cox) | 8 | 0.85 (0.70–1.03) | .09 | — |
| IVUS | Stent Thrombosis | IPD (Cox) | 16 | 0.54 (0.37–0.78) | .001 | — |
| IVUS | Stent Thrombosis | DL aggregate | 16 | 0.54 (0.36–0.81) | .003 | 0% |
| IVUS | TVR/TLR | IPD (Cox) | 16 | 0.70 (0.60–0.82) | <.001 | — |
| IVUS | TVR/TLR | DL aggregate | 16 | 0.65 (0.51–0.82) | <.001 | 44% |
| OCT | MACE | IPD (Cox) | 7 | 0.75 (0.63–0.91) | .003 | — |
| OCT | MACE | DL aggregate | 7 | 0.77 (0.64–0.92) | .004 | 0% |
| OCT | Cardiac Death | IPD (Cox) | 7 | 0.48 (0.27–0.83) | .009 | — |
| OCT | Cardiac Death | DL aggregate | 6 | 0.56 (0.33–0.97) | .037 | 0% |
| OCT | MI | IPD (Cox) | 7 | 0.79 (0.63–0.99) | .038 | — |
| OCT | MI | DL aggregate | 6 | 0.81 (0.65–1.00) | .055 | 0% |
| OCT | Stent Thrombosis | IPD (Cox) | 7 | 0.40 (0.22–0.74) | .004 | — |
| OCT | Stent Thrombosis | DL aggregate | 5 | 0.49 (0.27–0.89) | .019 | 0% |
| OCT | TVR | IPD (Cox) | 7 | 0.74 (0.57–0.97) | .029 | — |
| OCT | TVR | DL aggregate | 5 | 0.67 (0.40–1.12) | .129 | 54% |

CI, confidence interval; DL, DerSimonian–Laird; HR, hazard ratio; IPD, individual participant data; IVUS, intravascular ultrasound; MACE, major adverse cardiovascular events; MI, myocardial infarction; OCT, optical coherence tomography; RR, risk ratio; TLR, target-lesion revascularization; TVR, target-vessel revascularization; I², proportion of total variability due to between-study heterogeneity.

## Supplementary Table 2. Indirect comparison of IVUS vs OCT (subgroup meta-regression using angiography as common comparator)

| Outcome | IVUS HR (95% CI) | OCT HR (95% CI) | Ratio of HRs | P interaction |
| --- | --- | --- | --- | --- |
| MACE | 0.74 (0.67–0.83) | 0.75 (0.63–0.91) | 1.05 (0.84–1.32) | .88 |
| Cardiac Death | 0.79 (0.62–1.01) | 0.48 (0.27–0.83) | 1.75 (1.00–3.07) | .06 |
| Stent Thrombosis | 0.54 (0.37–0.78) | 0.40 (0.22–0.74) | 1.38 (0.66–2.91) | .55 |
| TVR/TLR | 0.70 (0.60–0.82) | 0.74 (0.57–0.97) | 0.99 (0.74–1.32) | .94 |

CI, confidence interval; HR, hazard ratio; IVUS, intravascular ultrasound; MACE, major adverse cardiovascular events; OCT, optical coherence tomography; TLR, target-lesion revascularization; TVR, target-vessel revascularization.

## Supplementary Table 3. Grambsch–Therneau global proportional-hazards test

| Endpoint | Modality | χ² | df | P | Interpretation |
| --- | --- | --- | --- | --- | --- |
| MACE | IVUS | 3.21 | 1 | .073 | No evidence against PH |
| Cardiac death | IVUS | 0.48 | 1 | .489 | PH assumption supported |
| TVR/TLR | IVUS | 2.07 | 1 | .150 | PH assumption supported |
| Stent thrombosis | IVUS | 0.92 | 1 | .337 | PH assumption supported |
| All-cause mortality | IVUS | 1.14 | 1 | .286 | PH assumption supported |
| MACE | OCT | 1.03 | 1 | .310 | PH assumption supported |
| Cardiac death | OCT | 0.22 | 1 | .640 | PH assumption supported |
| MI | OCT | 1.75 | 1 | .186 | PH assumption supported |
| Stent thrombosis | OCT | 0.14 | 1 | .709 | PH assumption supported |
| TVR | OCT | 4.28 | 1 | .039 | Borderline — time-varying effect plausible |

df, degrees of freedom; IVUS, intravascular ultrasound; MACE, major adverse cardiovascular events; MI, myocardial infarction; OCT, optical coherence tomography; PH, proportional hazards; TLR, target lesion revascularization; TVR, target vessel revascularization; χ², chi-squared statistic.

## Supplementary Table 4. Trial-Specific MACE Definitions, Follow-up Durations, and Imaging Optimization Protocols

| Trial | Year | FU (mo) | Composite Endpoint Definition | IVI Optimization Protocol |
| --- | --- | --- | --- | --- |
| IVUS-guided trials (n = 16) |  |  |  |  |
| HOME DES IVUS | 2010 | 18 | Death, MI, TLR | MSA ≥5.0 mm² (or CSA >90% of distal reference lumen CSA for small vessels); full apposition; no edge dissection |
| AVIO | 2013 | 24 | Cardiac death, MI, TVR | Post-dilation balloon sized to mean media-to-media diameter by IVUS; in-stent CSA below target considered underexpanded; criteria reduced 10% at stent edges |
| Kim JS 2013 | 2013 | 12 | CV death, MI, ST, TVR | IVUS-guided DES sizing and optimization for long lesions (≥28 mm) |
| AIR-CTO | 2015 | 24 | Death, cardiac death, MI, TLR, TVR | IVUS-guided CTO stenting; MSA >80% of reference vessel area; symmetry index >70%; no >Type B dissection |
| Tan 2015 | 2015 | 24 | Death, non-fatal MI, TLR | IVUS-guided stent selection and deployment in unprotected left main PCI |
| CTO-IVUS | 2015 | 12 | Cardiac death, MI, TVR | IVUS-guided CTO stenting; MSA ≥distal reference lumen area; stent area ≥5 mm² at CTO segment; complete apposition |
| Liu 2019 | 2019 | 12 | Cardiac death, MI, TVR | IVUS-guided DES implantation for unprotected left main lesions |
| IVUS-XPL | 2020 | 60 | Cardiac death, TL-MI, ID-TLR | IVUS criteria: MLA greater than distal reference lumen CSA (relative target); apposition confirmed |
| ULTIMATE | 2021 | 36 | Cardiac death, TV-MI, CD-TVR | IVUS criteria: MSA >5.0 mm² or ≥90% of distal-reference MLA; plaque burden <50% within 5 mm of edges; no edge dissection involving media |
| GUIDE-DES | 2023 | 12 | Cardiac death, TV-MI, ID-TLR | IVUS criteria: in-stent MLA greater than distal reference lumen CSA; complete apposition; no significant edge dissection |
| RENOVATE | 2023 | 24 | Cardiac death, TV-MI, CD-TVR | IVUS or OCT at operator discretion (~75% IVUS); MSA and apposition criteria per protocol |
| IVUS-ACS | 2024 | 12 | Cardiac death, TV-MI, CD-TVR | IVUS criteria: MSA >5.0 mm² or >90% of distal-reference MLA; plaque burden <55% within 5 mm of edges; no medial dissection >3 mm |
| DKCRUSH-VIII | 2026 | 12 | Cardiac death, TV-MI, CD-TVR | IVUS-guided bifurcation stenting; MSA criteria for both main vessel and side branch |
| IVUS-CHIP | 2026 | 12 | Cardiac death, TV-MI, CI-TVR | IVUS criteria: MSA >5 mm² or >90% of distal reference lumen; plaque burden <50% within 5 mm of edge; no edge dissection involving media >3 mm |
| OPTIMAL | 2026 | 12 | CV death, TV-MI, CI-TLR (DOC) | IVUS criteria: MLA ≥8 mm² (LM body), ≥7 mm² (polygon of confluence), ≥6 mm² (prox LAD), ≥5 mm² (prox LCx); minimal malapposition; no substantial edge dissection |
| Bendary 2024 | 2024 | 12 | MI, TVR, cardiac death | IVUS criteria: stent lumen CSA ≥6.9 mm²; complete stent apposition and expansion; no dissection |
| OCT-guided trials (n = 7) |  |  |  |  |
| OCTACS | 2015 | 6 | Death, MI, TLR | OCT criteria: underexpansion (MSA <90% of reference); malapposition (≥3 struts detached >140 µm); edge dissection; residual stenosis (MLA <4 mm²) |
| DOCTORS | 2016 | 6 | Death, MI, ST, TLR | OCT-guided PCI with post-dilation if underexpansion, malapposition, or edge dissection identified |
| Kala 2018 | 2018 | 9 | Death, MI, TLR | OCT criteria: MLA >80% of mean reference (or >90% of distal reference); no significant malapposition; no edge dissection |
| ILUMIEN IV | 2023 | 24 | Cardiac death, TV-MI, ID-TVR | OCT criteria: MSA ≥90% of reference; no malapposition >200 μm; no dissection flap ≥60° |
| OCTOBER | 2023 | 24 | Cardiac death, TL-MI, ID-TLR | OCT-guided PCI for complex bifurcation; assessment of stent expansion, apposition, and edge dissection |
| OCCUPI | 2024 | 12 | Cardiac death, MI, ST, ID-TVR | OCT-guided PCI for complex lesions; optimization criteria per protocol |
| CALIPSO | 2025 | 1 | CV death, MI, CD-TLR | OCT-guided PCI for calcified lesions; calcium modification guided by OCT findings |

ACS, acute coronary syndrome; CD-TLR, clinically driven target-lesion revascularization; CD-TVR, clinically driven target-vessel revascularization; CI-TLR, clinically indicated target-lesion revascularization; CI-TVR, clinically indicated target-vessel revascularization; CTO, chronic total occlusion; CV, cardiovascular; DES, drug-eluting stent; DOC, device-oriented composite; FU, follow-up; ID-TLR, ischemia-driven target-lesion revascularization; ID-TVR, ischemia-driven target-vessel revascularization; IVI, intravascular imaging; IVUS, intravascular ultrasound; MACE, major adverse cardiovascular events; MI, myocardial infarction; MLA, minimum lumen area; mo, months; MSA, minimum stent area; OCT, optical coherence tomography; PCI, percutaneous coronary intervention; ST, stent thrombosis; TL-MI, target-lesion myocardial infarction; TLR, target-lesion revascularization; TV-MI, target-vessel myocardial infarction; TVR, target-vessel revascularization.

## Supplementary Table 5. Leave-one-out sensitivity — IVUS MACE (DL random-effects)

| Analysis | Pooled RR | 95% CI |
| --- | --- | --- |
| Omitting CTO-IVUS | 0.68 | 0.55–0.85 |
| Omitting DKCRUSH-VIII | 0.69 | 0.56–0.86 |
| Omitting Tan 2015 | 0.68 | 0.54–0.85 |
| Omitting IVUS-XPL | 0.68 | 0.54–0.85 |
| Omitting ULTIMATE | 0.68 | 0.54–0.85 |
| Omitting IVUS-ACS | 0.68 | 0.54–0.85 |
| Omitting Liu 2019 | 0.67 | 0.53–0.84 |
| Omitting RENOVATE | 0.67 | 0.53–0.84 |
| Omitting Kim JS 2013 | 0.67 | 0.53–0.84 |
| Omitting AVIO | 0.66 | 0.52–0.83 |
| Omitting AIR-CTO | 0.65 | 0.52–0.82 |
| Omitting HOME DES IVUS | 0.66 | 0.52–0.82 |
| Omitting GUIDE-DES | 0.65 | 0.51–0.81 |
| Omitting OPTIMAL | 0.64 | 0.51–0.80 |
| Omitting IVUS-CHIP | 0.64 | 0.52–0.77 |
| Omitting Bendary 2024 | 0.69 | 0.56–0.86 |

CI, confidence interval; DL, DerSimonian–Laird; IVUS, intravascular ultrasound; MACE, major adverse cardiovascular events; RR, risk ratio.

## Supplementary Figure 1. Funnel plots for publication bias assessment of the primary endpoint (MACE) in the IVUS cohort (panel A; k = 16) and OCT cohort (panel B; k = 7). Each point represents one trial plotted against its standard error of log(HR). Dashed lines indicate the fixed-effect pooled estimate; shaded regions represent pseudo-95% confidence intervals. Neither cohort showed evidence of funnel-plot asymmetry (Egger’s regression test P > .05 for both).
